# Economic factors associated with county-level mental health – United States, 2019

**DOI:** 10.1371/journal.pone.0300939

**Published:** 2025-06-04

**Authors:** Michele L.F. Bolduc, Parya Saberi, Torsten B. Neilands, Carla I. Mercado, Shanice Battle Johnson, Zoe R. F. Freggens, Desmond Banks, Rashid Njai, Kai McKeever Bullard

**Affiliations:** 1 Office of Minority Health, Office of Health Equity, Centers for Disease Control and Prevention (CDC), Atlanta, Georgia, United States of America; 2 Division of Prevention Science, Department of Medicine, University of California, San Francisco, California, United States of America; 3 Division of Diabetes Translation, National Center for Chronic Disease Prevention and Health Promotion, CDC, Atlanta, Georgia, United States of America; University of Georgia, UNITED STATES OF AMERICA

## Abstract

A better understanding of whether and how economic factors impact mental health can inform policy and program decisions to improve mental health. This study looked at the association between county-level economic factors and the prevalence of self-reported poor mental health among adults in United States counties in 2019, overall and disaggregated for urban and rural counties. General dominance analyses were completed to rank-order the relative influence of the selected variables in explaining county prevalence of adults reporting > 14 poor mental health days in the last 30 days (“poor mental health”). The highest weighted variables were assessed for the statistical significance of their relationships with county-level poor mental health through multiple linear regression. Across all models, the four highest-ranked economic factors were household income, receipt of Supplemental Security Income, population with a college degree, and receipt of Supplemental Nutrition Assistance Program benefits. The overall, rural, and urban models explained over 68% of the variation in poor mental health prevalence between counties. Urban and rural models showed notable differences in the top factors associated with poor mental health and opposite associations between poor mental health and population with public insurance. The findings from this study indicate a significant association between several economic factors and poor mental health, which may inform decision makers in addressing mental health in the United States.

## Introduction

Mental health is a key component of overall health and well-being, and mental health conditions are common in the United States [1]. The prevalence of any mental, behavioral, or emotional disorder among US adults has been increasing since 2008, reaching 20.6% by 2019 [2]. An estimated 9.2% of adults have received mental health treatment in the last 12 months [3], but almost half (43.8%) of adults who needed treatment did not receive it [2]. Poor mental health, which includes stress, depression, and problems with emotions, can be experienced by people with or without a diagnosable mental health condition. It is a risk factor for a range of chronic diseases and injuries, from stroke to diabetes [4–6]. Poor mental health is also costly for the economy in terms of losses of productivity and the costs of treatment [7]. For example, Major Depressive Disorder alone cost the US an estimated $326 billion in 2018, a 37.9% increase over the costs in 2010, primarily due to increasing workplace costs [8]. Though the causes of poor mental health are complex, addressing the preventable factors that affect mental health could reduce the economic burden of poor mental health and improve overall public health.

The socio-ecological model of mental health acknowledges the role that individual, social, and societal factors have in mental health and well-being [9–11]. These factors, visualized by Michaels et al 2021 as six levels of interacting and nested social and environmental factors [10], may have a positive or negative role in the development or worsening of poor mental health. One such factor is an individual’s socioeconomic and financial situation, including employment status and income. One survey found that among people in the US, the top personal stressors were work (64% of respondents) and money (60%); 46% indicated the economy was a significant source of stress [1]. Exposure to adverse conditions, such as material hardship, “undoubtedly leads to stress and known psychological and physiological stress responses” that increase the risk of mental health conditions [12]. These issues may be exacerbated on a population level in difficult economic times, with higher rates of suicide, homicide, substance abuse, and psychiatric disorders noted during financial crises [13–16]. An exploration of the economic factors that impact mental health could inform possible public health interventions.

Improving population mental health requires a broadening of focus from individual treatment – which is costly to patients and the healthcare system – to population-level or systems-based approaches. Specifically, it necessitates a move from the more proximal non-medical factors that impact health to the upstream drivers of those factors [17–19]. Upstream drivers relate to how policy and economics shape the distribution of resources; changes at this level have the potential for greater impacts on population health [17,20]. Upstream interventions in policy, programs, and practice place the responsibility of health on decision-makers to undertake broader systemic change rather than focus on individual choice and behavior [12], which could have larger impacts across the life course and across generations [21].

Data demonstrating whether economic factors may be associated with mental health can inform policy and program decisions. This study, therefore, examines the association between county-level economic variables impacting health (referred to in this paper as ‘economic factors’) and the prevalence of self-reported poor mental health among adults in US counties in 2019.

## Methods

This analysis is a cross-sectional, ecological study of the association between county-level economic variables (e.g., county Gross Domestic Product [GDP], income inequality, housing affordability) and self-reported poor mental health prevalence for US counties in 2019. Data for 2019 were used to get a baseline understanding of the economic factors associated with mental health prior to the start of the COVID-19 pandemic.

The dependent variable of self-reported poor mental health comes from Centers for Disease Control and Prevention (CDC) PLACES data (www.cdc.gov/places), which produces small area estimates using data from CDC’s Behavioral Risk Factor Surveillance System (BRFSS). The BRFSS (www.cdc.gov/brfss) is a telephone survey through which states collect information on health-related risk behaviors and health conditions, with over 400,000 adult participants each year. The survey asks the question, “Now thinking about your mental health, which includes stress, depression, and problems with emotions, for how many days during the past 30 days was your mental health not good? [22]” BRFSS defines poor mental health as responses indicating >14 poor mental health days over the last 30 days. PLACES uses the BRFSS data to generate county-level prevalence estimates of poor mental health for all US states and the District of Columbia, using a multilevel statistical model that considers individual-level demographic data from BRFSS, county-level percentage of adults below 150% of the federal poverty level from the American Community Survey (ACS), and state- and county-level random effects [23]. Estimates for poor mental health prevalence are available for 3,121 counties for 2019, with 23 missing counties in New Jersey (not available from BRFSS) and Alaska (missing the newer Chugach and Copper River Census Areas).

Economic variables originate from the US Bureau of Economic Analysis (BEA) and from American Community Survey (ACS) five-year estimates for 2019 (S1 Table). Variables were chosen to measure economic factors (relating to the production, distribution, and consumption of goods and services) meaningful at the county level. Business variables from BEA were selected to measure current and changing county-level economic activity, including real GDP, 10-year change in GDP, and 10-year change in county GDP. Business variables from BEA are not available for 51 jurisdictions in Virginia due to combined independent city/county estimates, nor for two counties in Hawaii (Maui and Kalawao) due to combined county estimates. Employment variables covered a range of work-related issues, including unemployment rate, employed but under the Federal Poverty Level (known throughout this paper as ‘working poverty rate’), mean usual hours worked over the last 12 months, employees working from home, and mean travel time to work. Income and wealth variables include median individual earnings, median household income, population with a college degree, two measures of inequality (Gini Index of income inequality and the female/male pay gap), percent of the population with public assistance income (Social Security, Supplemental Security Income [SSI], and cash public assistance), as well as homeownership rate (as a significant component of wealth-building). Several variables were included to cover key expenses – two housing affordability variables (for homeowners, population with Selected Monthly Owner Costs as a Percentage of Household Income [SMOCAPI] costs of at least 30%, and, for renters, population with Gross Rent as a Percent of Household Income [GRAPI] costs of at least 30%), a food security variable (percent receiving Supplemental Nutrition Assistance Program or SNAP benefits), and two health insurance coverage variables (percent uninsured and percent with public health insurance coverage). Two more variables were added to capture county-level economics, including median home value and 10-year population change. Two additional variables from County Health Rankings (CHR) were used to capture the county availability of healthcare providers, which could shape the prevalence of poor mental health through availability of medical treatment: Primary Care Provider Ratio (from the CHR 2022 dataset) and Mental Health Provider Ratio (from the CHR 2020 dataset), both of which indicate the ratio of the population to the number of health providers. All data were merged into a single dataset using Federal Information Processing Standards (FIPS) codes for each county as the common attribute.

Our first step was to use PLACES data to map the prevalence of poor mental health at the county level for 2019 in ArcGIS Pro 2.9.7 (Environmental Systems Research Institute, Redlands, CA, USA). Stata version 17.0 (StataCorp LP, College Station, TX, USA) was used to complete the following analyses for all US counties and by county urban or rural classification based on the 2013 USDA Rural-Urban Continuum Codes. The median and Interquartile Range (IQR) for each variable were described across all counties in the datasets. Next, variable clustering was used to remove two highly collinear (>0.5 Spearman coefficient) and redundant (based on our understanding of the literature) variables from our analysis: median earnings (keeping median household income) and change in real GDP (keeping change in GDP). General dominance analyses were then completed using the Stata package *domin* with all the remaining economic variables to rank-order the relative influence of the variables in explaining county-level poor mental health, overall and for urban and rural counties separately [24]. This method finds the proportion of the total R^2^ accounted for by each variable based on the average R^2^ across all possible subsets of regression models. Using scree plots (S1 Fig), the most influential (highest weighted) variables were identified from the dominance analyses based on dominance weights above the “elbow” of the graph. We retained eleven influential variables in the overall model, seven in the urban model, and eight in the rural model. The retained variables were then assessed for the statistical significance of their relationships with county-level poor mental health through multiple linear regression.

## Results

**Fig 1:**
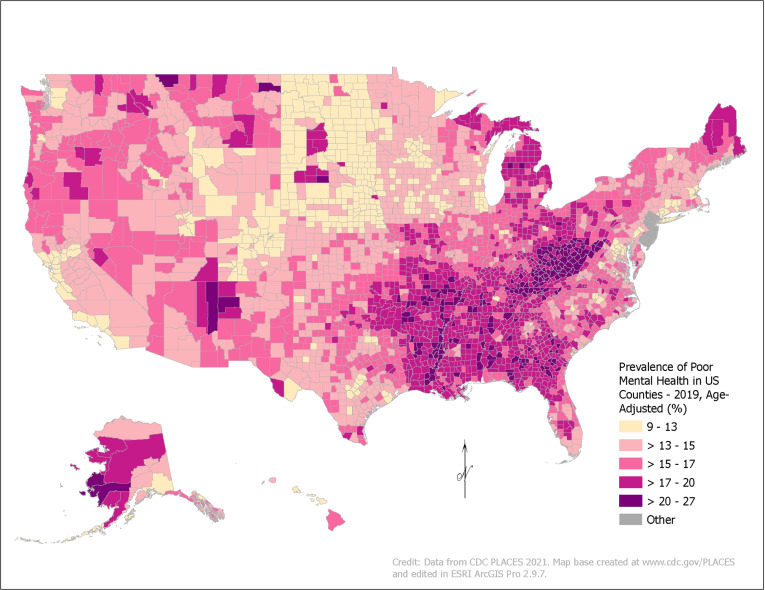
Estimated Prevalence of Poor Mental Health by County, United States, 2019. Data come from CDC PLACES, which is modeled from the CDC Behavioral Risk Factor Surveillance System (BRFSS). BRFSS asks the question, “Now thinking about your mental health, which includes stress, depression, and problems with emotions, for how many days during the past 30 days was your mental health not good?” Self-reported poor mental health is defined as responses of >14 poor mental health days over the last 30 days.

Fig 1 shows the prevalence of poor mental health across all counties in 2019, with a mean of 16.0% and a median of 15.8% (IQR: 14.1–17.8%). County prevalence ranged from 9.7% (Falls Church, VA) to 26.3% (East Carroll Parish, LA). Across the US, prevalence was higher in Appalachia and the Deep South and parts of Alaska, Montana, South Dakota, and the Southwest. Lower prevalence was found in the Upper Midwest.

Median and IQR values for each economic variable in 2019 are listed in Table 1. Table 2 shows the most influential economic variables for county-level poor mental health identified using the dominance analysis based on standardized dominance weights. The top variables for all counties overall were percent of households with SSI, median household income, percent of households receiving SNAP benefits in the last 12 months, percent of the population 25 years or older with a college degree, and percent of the population with public health insurance. The top results were similar for urban counties. For rural counties, the results were similar, but travel time from work ranked higher than receipt of public insurance.

**Table 1:** Summary Statistics for Variables Used to Analyze County-Level Association Between Economic Factors and Estimated Prevalence of Poor Mental Health, United States, Overall and by Urban/Rural Classification, 2019.

Factor	Variable	2019 Median [IQR]		
		Overall N = 3,121	Urban n = 1,145	Rural n = 1,976
Business	10-year percent change in Gross Domestic Product (GDP), 2010–2019 (%)	28.2% [15.3 – 42.8%]	35.6% [22.6 – 49.2%]	24.6% [10.9 – 39.1%]
	Real GDP, in thousands of chained dollars ($)	$946,103 [$361,013 – $2,691,786]	$4,018,925 [$1,023,404 – $10,900,000]	$595,270 [$263,664 – $1,295,594]
	Change in Real GDP, 2010–2019 (%)	9.7% [-1.8 – 22.3%]	14.1% [3.6 – 26.3%]	6.4% [-4.38 – 19.5%]
Employment	Unemployment rate, ≥ 16 years old (%)	4.9% [3.6 – 6.5%]	4.9% [3.9 – 6.1%]	5.0% [3.4 – 6.8%]
	Employed but under the Federal Poverty Limit (FPL) (%)	6.8% [5.1 – 8.9%]	6.0% [4.4 – 7.9%]	7.3% [5.5 – 9.3%]
	Mean usual hours per week worked in the past 12 months for workers 16–64 years old	39.3 hours [38.5 – 40.2 hours]	39.0 hours [38.3 – 39.7 hours]	39.6 hours [38.6 – 40.7 hours]
	Employees ≥ 16 years old working from home (%)	4.5% [3.2 – 6.3%]	4.5% [3.4 – 5.8%]	4.4% [3.0 – 6.7%]
	Mean travel time to work (minutes)	23.7 minutes [19.8 – 27.5 minutes]	25.7 minutes [22.4 – 29.5 minutes]	22.4 minutes [18.4 – 26.0 minutes]
Income and Wealth	Median earnings ($)	$32,607 [$30,660 – $36,916]	$35,963 [$32,175 – $40,070]	$31,738 [$29,801 – $34,995]
	Median household income ($10,000 increments)	$5.2 [$4.4 – $6.0]	$5.8 [$5.1 – $6.8]	$4.9 [$4.2 – $5.5]
	Population ≥ 25 years old with a college degree (%)	19.5% [15.3 – 25.8%]	24.8% [18.6 – 33.1%]	17.8% [14.3 – 22.1%]
	Gini Index of income inequality (index 0–1)	0.44 [0.42 – 0.47]	0.44 [0.42 – 0.47]	0.44 [0.42 – 0.47]
	Female pay as a percentage of male pay (%)	76.5% [71.4 – 81.8%]	78.0% [73.4 – 82.3%]	75.8% [70.2 – 81.3%]
	Households with Social Security income (%)	37.4% [32.8 – 41.8%]	33.8% [29.4 – 37.9%]	39.2% [35.3 – 43.3%]
	Households with Supplemental Security Income (SSI) (%)	5.5% [4.1 – 7.3%]	5.1% [4.0 – 6.6%]	5.8% [4.2 – 8.0%]
	Households with public cash assistance (%)	1.9% [1.3 – 2.8%]	1.9% [1.4 – 2.6%]	1.9% [1.3 – 2.9%]
	Homeownership (%)	72.9% [67.6 – 77.2%]	71.7% [64.6 – 77.0%]	73.4% [68.9 – 77.2%]
Expenses	Prevalence of units with a mortgage with Selected Monthly Owner Costs as a Percentage of Household Income (SMOCAPI) of 30% or more (%)	24.6% [20.9 – 29.4%]	24.2% [21.2 – 28.5%]	24.8% [20.6 – 29.8%]
	Prevalence of rent-paying units with Gross Rent as a Percentage of Household Income (GRAPI) of 30% or more (%)	44.3% [38.1 – 49.5%]	46.6% [42.3 – 50.8%]	42.4% [35.8 – 48.4%]
	Households with Supplemental Nutrition Assistance Program (SNAP) benefits in the past 12 months (%)	11.9% [8.3 – 16.2%]	11.1% [8.0 – 14.6%]	12.6% [8.5 – 17.1%]
	Population 19–64 years old without health coverage (%)	8.7% [5.8 – 12.1%]	8.0% [5.3 – 10.8%]	9.1% [6.1 – 13.2%]
	Population with public health insurance coverage alone (Medicaid, Medicare, Veterans Administration [VA]) (%)	39.4% [33.2 – 45.3%]	35.6% [30.3 – 41.3%]	41.7% [35.7 – 47.3%]
Neighborhood	Median home value, in $10,000 increments ($)	$12.7 [$9.8 – $17.4]	$16.7 [$13.0 – $22.6]	$11.0 [$8.8 – $14.4]
	10-year population change (%)	-0.07% [-3.6 – 5.2%]	4.3% [-0.35 – 10.5%]	-1.8% [-4.9 – 2.0%]
Health Provider Supply	Ratio of population to number of primary care providers	2055.9 [1343.6 – 3084.3]	1772.5 [1216.7 – 2795.6]	2187.3 [1479.7 – 3251.3]
	Ratio of population to number of mental health providers	786.4 [408.4 – 1747.9]	615.8 [348.3 – 1270.0]	958.3 [465.3 – 2128.3]
Dependent Variable	Prevalence of > 14 poor mental health days of the last 30 days, age-adjusted (%)	15.8% [14.1 – 17.8%]	15.3% [13.7 – 16.9%]	16.1% [14.4 – 18.4%]

NB: The dependent variable is missing 21 counties in New Jersey and 2 counties in Alaska, limiting the overall analysis to N = 3,121 counties (urban n = 1,145; rural n = 1,976). The three Business variables are missing 2 counties in Hawaii and 51 counties in Virginia (Overall Analytic N = 3,068; Urban n = 1,109; Rural n = 1,959). The primary care provider ratio is missing 153 counties (N = 2,968; Urban n = 1,118; Rural n = 1,850). The mental health provider ratio is missing 204 counties (N = 2,917; Urban n = 1,116; Rural n = 1,801).

**Table 2:** Most-to-Least Influential County-Level Economic Variables Contributing to County Prevalence of Poor Mental Health Based on Dominance Analysis Ranked by Standardized Dominance Weights, Overall and by Urban/Rural Classification, United States, 2019.

2019 Dominance Analysis Rankings and Weights					
Overall N = 2,772	Weights	Urban n = 1,061	Weights	Rural n = 1,711	Weights
1. Household income	0.1136	1. Household income	0.1157	1. Household income	0.1160
2. Receipt of SSI	0.1126	2. Receipt of SSI	0.1090	2. College degree	0.1096
3. College degree	0.0952	3. College degree	0.0834	3. Receipt of SSI	0.1072
4. Receipt of SNAP	0.0944	4. Receipt of SNAP	0.0833	4. Receipt of SNAP	0.0899
5. Public insurance	0.0815	5. Public insurance	0.0768	5. Work travel time	0.0841
6. Unemployment	0.0652	6. Social Security	0.0763	6. Public insurance	0.0758
7. Work from home	0.0607	7. Home values	0.0693	7. Unemployment	0.0739
8. Home values	0.0597	8. Work from home	0.0501	8. Work from home	0.0677
9. Social Security	0.0580	9. Working poverty	0.0470	9. Working poverty	0.0475
10. Work travel time	0.0521	10. Unemployment	0.0388	10. Home values	0.0440
11. Working poverty	0.0520	11. Work travel time	0.0305	11. Social Security	0.0389
12. Gini Index	0.0280	12. Population change	0.0299	12. Gini Index	0.0346
13. Uninsured	0.0185	13. SMOCAPI ≥30%	0.0265	13. GRAPI ≥30%	0.0213
14. Population Change	0.0181	14. Real GDP	0.0219	14. Uninsured	0.0151
15. PCP Ratio	0.0147	15. F/M pay gap	0.0197	15. PCP Ratio	0.0127
16. Real GDP	0.0144	16. Uninsured	0.0193	16. Hours worked	0.0120
17. Homeownership	0.0121	17. Homeownership	0.0192	17. Homeownership	0.0100
18. GRAPI ≥30%	0.0106	18. Gini Index	0.0189	18. SMOCAPI ≥30%	0.0095
19. SMOCAPI ≥30%	0.0099	19. Change GDP	0.0187	19. Cash assistance	0.0080
20. Cash assistance	0.0074	20. PCP Ratio	0.0152	20. Real GDP	0.0076
21. Change GDP	0.0066	21. Cash assistance	0.0101	21. Population change	0.0057
22. Hours worked	0.0059	22. MHP Ratio	0.0083	22. Change GDP	0.0034
23. MHP Ratio	0.0049	23. GRAPI ≥30%	0.0062	23. MHP Ratio	0.0032
24. F/M pay gap	0.0038	24. Hours worked	0.0057	24. F/M pay gap	0.0022

* SSI = Supplemental Security Income, SNAP = Supplemental Nutrition Assistance Program, PCP = Primary Care Provider, GDP = Gross Domestic Product, GRAPI = Gross Rent as a Percent of Household Income, SMOCAPI = Selected Monthly Owner Costs as a Percentage of Household Income, MHP = Mental Health Provider, F/M = Female/Male.

In the overall model using the top eleven variables identified in the scree plot, county-level measures significantly positively associated with the prevalence of poor mental health included: unemployment, working poverty rate, mean travel time to work, households with Social Security, households with SSI, and households with SNAP benefits (Table 3). County-level measures significantly inversely associated with poor mental health prevalence were employees working from home, median household income, and percent of the population with a college degree. The largest coefficients were in median household income, percent of the population working from home, and population receiving SSI. The variables in the overall model explained 70% (Adjusted R^2^ = 0.7001) of the variation in poor mental health prevalence between counties.

**Table 3:** Beta Coefficients, 95% Confidence Interval, and Statistical Significance for County-Level Economic Variables Using Linear Regression with Prevalence of Poor Mental Health as the Dependent Variable, Overall and by Urban/Rural Classification, United States, 2019. Blue-filled cells indicate a positive association between the variable and the dependent variable; red-filled cells indicate a negative association; greyed out cells indicate the variable was not significant; blank cells indicate a variable that was not included in the model.

Factor	Variable	2019		
		Overall N = 3,121	Urban n = 1,145	Rural n = 1,976
Employment	Unemployment rate, 16 years old + (%)	**0.093** **(0.066, 0.12)** **p < 0.001**		**0.11** **(0.076, 0.14)** **p < 0.001**
	Employed but under the Federal Poverty Level (FPL) (%)	**0.055** **(0.031, 0.078)** **p < 0.001**		
	Employees aged 16 years and older working from home (%)	**-0.12** **(-0.14, -0.10)** **p < 0.001**		**-0.11** **(-0.13, -0.093)** **p < 0.001**
	Mean travel time to work (minutes)	**0.085** **(0.075, 0.095)** **p < 0.001**		**0.080** **(0.067, 0.093)** **p < 0.001**
Income and Wealth	Median household income ($10,000 increments)	**-0.58** **(-0.67, -0.48)** **p < 0.001**	**-0.40** **(-0.50, -0.30)** **p < 0.001**	**-0.77** **(-0.87, -0.67)** **p < 0.001**
	Population 25 years old and older with a college degree (%)	**-0.037** **(-0.046, -0.028)** **p < 0.001**	**-0.036** **(-0.047, -0.024)** **p < 0.001**	**-0.049** **(-0.060, -0.037)** **p < 0.001**
	Households with Social Security income (%)	**0.024** **(0.012, 0.035)** **p < 0.001**	**0.072** **(0.054, 0.091)** **p < 0.001**	
	Households with Supplemental Security Income (SSI) (%)	**0.13** **(0.096, 0.16)** **p < 0.001**	**0.31** **(0.24, 0.37)** **p < 0.001**	**0.087** **(0.052, 0.12)** **p < 0.001**
Expenses	Households with Supplemental Nutrition Assistance Program (SNAP) benefits in the past 12 months (%)	**0.030** **(0.013, 0.047)** **p = 0.0014**	**0.067** **(0.037, 0.097)** **p < 0.001**	**0.031** **(0.012, 0.051)** **p = 0.002**
	Population with public health insurance coverage alone (Medicaid, Medicare, Veterans Administration [VA]) (%)	0.0083 (-0.0040, 0.021) p = 0.18	**-0.039** **(-0.061, -0.017)** **p = 0.001**	**0.025** **(0.014, 0.037)** **p < 0.001**
Neighborhood	Median home value ($10,000 increments)	-0.0042 (-0.014, 0.0051) p = 0.38	**-0.020** **(-0.032, -0.0087)** **p = 0.001**	
**Adj R**^**2**^ **Value**		**0.7001**	**0.6867**	**0.6914**

For urban counties, all seven variables used in the model were highly statistically significant in similar directions; however, two variables that were not significant in the overall model were negatively associated with poor mental health in urban counties: population with public insurance and median home value. The model explained over 68% (Adjusted R^2^ = 0.6867) of the county variation in poor mental health prevalence.

In the rural model, all eight county-level economic measures used in the regression were statistically significantly associated with poor mental health prevalence, in the same direction as the associations in the overall model. In contrast to urban counties, public health insurance was significantly *positively* associated with poor mental health. The adjusted R^2^ values indicate that the rural model explained 69.1% of the variation in county-level prevalence of poor mental health.

## Discussion

We identified the top most influential economic factors associated with self-reported poor mental health at the US county level; these variables explained at least 68% of the variation in poor mental health between counties in 2019 in all models. The explanatory variables associated with poor mental health were consistent in urban and rural models with some variation in their weighted ranking in the dominance analysis. Across all models, the four highest-ranked economic factors were related to income and expenses: receipt of SSI, household income, receipt of SNAP benefits, and population with a college degree.

Based on our findings, median household income was the most influential factor associated with county-level poor mental health in all models. Our results show that higher median income in a county is associated with a lower prevalence of poor mental health. Income is a key factor in overall health because it provides households with the resources necessary for health and wellbeing, including basic needs like nutritious food, safe housing, and health care services and other resources like higher education [25]. Lower incomes put children and adults at higher risk for stress, worse physical health, exposure to adverse conditions in early life, and exposure to violence and crime, any of which could trigger mood and anxiety disorders [26–28]. A reduction in or loss of income may be particularly harmful to mental health [26,29,30]. In addition, there is evidence that interventions aimed at reducing poverty, such as through cash transfers, have a positive effect on mental health, particularly when the support lifts someone out of poverty [26,29].

One type of income support, SSI benefits, was also significant in all models. The results showed that as the percentage of the population with SSI benefits increased, so too did the prevalence of poor mental health. This relationship is interpreted as bidirectional: receipt of SSI could be an economic indicator that influences mental health, but mental health conditions may enable someone to receive SSI. SSI provides fixed, monthly supplemental financial assistance to people who are: under 65 years old who are blind or have a disability (including mental disorders) that makes it difficult to perform substantial gainful activity or over 65 years old without a disability who live on a low income. Among the 72% of SSI recipients who are under 65 years old, six out of 10 were receiving SSI due to a mental health disorder [31]. In addition, adults with disabilities or who have low incomes are more likely to report frequent mental distress than those without disabilities or with higher incomes [32]. The significant association between receipt of SSI and prevalence of poor mental health could be explained by the higher rate of mental health disorders among SSI recipients than among the general public; however, the relationship may also suggest that the program on its own may not be able to economically mediate the impact that other factors have on the number of poor mental health days experienced by people with disabilities (including mental illness) and/or low incomes.

Similarly, a higher number of SNAP recipients in a county was associated with higher prevalence of poor mental health. To be eligible for SNAP benefits, households must meet certain gross and net income and resource limits, making SNAP a proxy for low-income households. Under special SNAP rules, recipients with disability income (e.g., SSI) only need to meet net income limits. The positive relationship between SNAP benefits and poor mental health may reflect higher prevalence of mental health conditions among recipients. An estimated 28% of non-elderly SNAP recipients have a disability (which could include mental health conditions); of those with a disability, almost 60% receive social security benefits [33]. Though SNAP benefits may have a positive impact on public health [34], the results of these models suggest that SNAP benefits may not be able to make up for the financial gaps people with low incomes and/or disabilities may have and the impacts of these financial shortfalls on mental health.

Although the percentage of the population with public health insurance coverage was a highly weighted factor in the dominance analysis for all models, it was not statistically significant in the overall model and opposite relationships were seen in the urban (negative) and rural (positive) models. Like SSI and SNAP households discussed previously, the positive relationship between public health insurance coverage and poor mental health may indicate that access to public insurance may not mediate the impact of having a low income, and this appears to be particularly true for rural areas. Having public health insurance may play more of a protective role in the prevalence of poor mental health in urban counties. The contrasting relationships to poor mental health in urban and rural counties support the need to examine associations with urban and rural economic factors separately.

The significance of having a college degree for lower prevalence of poor mental health in all models may be related to higher wages associated with greater educational attainment [35], access to jobs with benefits like health coverage, and the increased potential to build wealth with a college degree. College education may also have an independent effect on mental health after controlling for income due to increased non-income related benefits, like broader social networks and other resources, resulting in lower rates of death and longer lives [12]. Lower levels of educational attainment are associated with increased risk of some psychiatric diagnoses like Major Depressive Disorder and Generalized Anxiety Disorder [36] and higher rates of suicide [37]. Conversely, there is a bidirectional relationship between education and mental health: poor mental health is associated with higher risk of early termination of education, limiting educational attainment [38].

In addition to the income and expense variables, we found that as mean travel time to work (used in the overall and rural models) increased, the prevalence of poor mental health increased, which could be explained as a function of the increased time spent making driving-related decisions, leading to higher stress levels [39]. The results showed that the percentage of employees working from home (used in the overall and rural models) was inversely associated with poor mental health, which also may be related to commute time and other aspects of remote work that support mental well-being, such as fewer distractions, a more comfortable environment, and more time to spend with family or cook meals [40].

An unexpected result was the null finding for median home values across all US counties, yet significant association for the urban model. Median home value in urban counties had a significant inverse relationship with poor mental health. This is consistent with other studies like those on the Moving to Opportunity for Fair Housing Demonstration Program that have shown that higher income and higher opportunity neighborhoods may improve mental and physical health, especially for adults [41–43], possibly relating to less exposure to neighborhood level stressors like crime [43] and greater access to health-promoting resources like high-quality medical providers [44] and recreation facilities and amenities in good condition [45] than are available in poorer neighborhoods.

While individual- and clinical-level interventions to address these economic drivers exist, such as connecting patients receiving mental health care with economic resources to ease financial burdens, these types of interventions are not sufficient [12]. Population health improves as income increases, particularly for populations in the lowest income brackets [26,29]. Interventions that systematically address poverty and the financial burden of basic needs like health insurance and food may have a greater impact on health in the US than a narrow focus on individual interventions [20]. Addressing the economic drivers of poor mental health may also have cascading impacts on a range of other chronic health outcomes that share the same drivers, from mortality to substance abuse [46–49].

Policy considerations relating to household income that may have a positive effect on county-level mental health include expanding programs for federal income subsidies [50]; ensuring income support for households during difficult financial times, such as unemployment [51,52]; establishment of a universal basic income [53]; increasing federal, state, or local minimum wages [54,55]; offering additional tax breaks or credits for low-income households [56,57]; and enacting policies which promote broader access to higher-paying employment opportunities [58]. Programs like SSI that provide financial assistance to people with poor mental health who may have challenges maintaining stable work and health insurance for treatment could positively impact mental health. Similarly, policies and programs that support people with low incomes so they can afford necessary expenses like housing and food may have a beneficial impact on mental health, beyond the original intentions of the program [59,60]. Additional interventions that might benefit population mental health include increasing educational opportunities, such as high school completion initiatives, low-cost technical college programs, and college tuition assistance [61,62]; expanding access to healthcare through subsidized or universal health insurance coverage [63–65]; and thoughtful implementation of policies supporting work from home [66].

Future analyses could evaluate which approaches are most likely to improve mental health. However, economic considerations may be necessary but insufficient to comprehensively address the persistence of poor mental health in the US. A systems approach would help understand the most effective ways to improve mental health. Impactful changes in one place can have cascading effects and create systemic change [21]. Assessment of the mental health consequences of any new policy or policy change could be standard practice, in line with a Health in All Policies approach [67]. However, the full effects of large-scale, upstream, systemic changes may not be seen for many years [7].

Additional research could clarify the relationships between economic factors and mental health at the individual level, but more data on non-medical factors (such as standardized data in electronic health systems [68]) are needed to capture how mental health may be influenced by non-medical factors on an individual basis. Relatedly, the importance of each economic variable would be expected to vary depending on other geographic and demographic variables (e.g., age, sex, race, ethnicity, and nativity). Social and economic inequalities persist for various demographic groups [69], and these issues are not fully reflected in existing datasets. Future research could continue to explore the relationships between economic factors and other variables that indicate differences in health outcomes across populations and places, as well as the most effective change points for interventions.

Our study had three main limitations. First, we conducted an ecological, cross-sectional study to assess county-level associations, limiting our ability to infer causality. To minimize bias and the influence of confounding, future studies could employ sophisticated models such as fixed effects with time-dependent predictors using longitudinal data. For example, researchers could explore how changes in any of these factors may lead to short- or long-term changes in mental health outcomes. Second, this study carries forward the limitations of our original data sources. For example, the mental health prevalence estimates use data from BRFSS, which uses self-reported answers to a single question to assess mental health status. The variable is broad and likely includes a range of mental health conditions, from diagnosable mental illness to stress, that impact number of poor mental health days. We expect these conditions have varying relationships to the economic factors explored here. Additional studies could explore the associations between economic factors and the prevalence of specific mental health conditions and/or alternative measures of poor mental health. Third, reduced sample sizes in the urban- and rural-specific models may have impacted our findings.

Nevertheless, this study contributes to the existing literature on the economics of poor mental health in three important ways. First, we explore a range of economic factors at the county level that may influence mental health status, including unique neighborhood-level and economic variables that could be used as a starting point for further analysis. It also provides a baseline analysis to understand economic factors related to mental health at the county-level prior to the major economic disruptions associated with the COVID-19 pandemic, which worsened mental health challenges [70–72]. Finally, it applies dominance analysis techniques to go beyond the identification of potential drivers to rank these drivers in order of influence, which can inform the prioritization of policies and programs to improve mental health status.

## Conclusions

This study provides important data to researchers and policymakers on the association of economic factors and mental health. This study found that higher county-level access to resources, including income, education, and health insurance, is associated with a lower prevalence of poor mental health. Economic factors shape the distribution of resources and opportunities needed to maintain mental well-being and these distributions are geographically uneven across the US. Additional research could explore additional economic variables as indicators and their relationship to specific mental health outcomes (e.g., depression); spatial trends in poor mental health over time; and possible mental health-specific population-level interventions and their effectiveness in improving mental health to improve public health and address geographic and demographic differences in negative mental health outcomes.

## Supporting information

S1 Fig Scree Plots of Dominance Weights Identified by the Dominance Analysis of Economic Variables in Relation to Poor Mental Health, for Overall, Urban, and Rural Counties. (a) In the overall model, the “elbow” on the plot at variable 12 (indicated in red) shows where the dominance weights drop off most steeply in value; values to the left of the elbow (11 total) were retained as significant for our overall linear regression model. (b) The urban scree plot indicates a steep drop in weights after variable 2, and the second steepest drop after variable 7. To capture additional factors impacting mental health, we retained the top seven variables for the urban linear regression model. (c) The steepest decline in the rural scree plot came after variable 8. The top eight variables were retained for the rural linear regression model.
(DOCX)

S1 Table Variables Used to Analyze County-Level Association Between Economic Factors and Estimated Prevalence of Poor Mental Health and their Sources, United States, 2019. (DOCX)

## References

[1] American Psychological Association. Stress in America: Stress and current events. 2019. Available from: https://www.apa.org/news/press/releases/stress/2019/stress-america-2019.pdf

[2] Substance Abuse and Mental Health Services Administration. Key substance use and mental health indicators in the United States: Results from the 2019 National Survey on Drug Use and Health. HHS Publication No PEP20-07-01-001, NSDUH Series H-55. 2020. Rockville, MD: Center for Behavioral Health Statistics and Quality. Available from: https://www.samhsa.gov/data/report/2019-nsduh-annual-national-report

[3] Terlizzi EP, Schiller JS. Mental health treatment among adults aged 18-44: United States, 2019-2021. NCHS Data Brief, No 444. 2022. Hyattsville, MD: National Center for Health Statistics. Available from: 10.15620/cdc:120293 36135999

[4] Liao B, Xu D, Tan Y, Chen X, Cai S. Association of mental distress with chronic diseases in 1.9 million individuals: A population-based cross-sectional study. J Psychosom Res. 2022;162:111040. doi: 10.1016/j.jpsychores.2022.111040 36137487

[5] Rashid M, Mazharul Islam M, Li A, Shifa N. Frequent mental distress among adults in the United States and its association with socio-demographic characteristics, lifestyle, and chronic health condition. JPHD. 2022;20(1). doi: 10.55131/jphd/2022/200112

[6] Scott KM, Lim C, Al-Hamzawi A, Alonso J, Bruffaerts R, Caldas-de-Almeida JM, et al. Association of Mental Disorders With Subsequent Chronic Physical Conditions: World Mental Health Surveys From 17 Countries. JAMA Psychiatry. 2016;73(2):150–8. doi: 10.1001/jamapsychiatry.2015.2688 26719969 PMC5333921

[7] Knapp M, Wong G. Economics and mental health: the current scenario. World Psychiatry. 2020;19(1):3–14. doi: 10.1002/wps.20692 31922693 PMC6953559

[8] Greenberg PE, Fournier A-A, Sisitsky T, Simes M, Berman R, Koenigsberg SH, et al. The Economic Burden of Adults with Major Depressive Disorder in the United States (2010 and 2018). Pharmacoeconomics. 2021;39(6):653–65. doi: 10.1007/s40273-021-01019-4 33950419 PMC8097130

[9] Reupert A. A socio-ecological framework for mental health and well-being. Advances in Mental Health. 2017;15(2):105–7. doi: 10.1080/18387357.2017.1342902

[10] Michaels C, Blake L, Lynn A, Greylord T, Benning S. Mental health and well-being ecological model. Center for Leadership Education in Maternal & Child Public Health, University of Minnesota–Twin Cities. 2021. Retrieved August 28, 2024 from https://mch.umn.edu/resources/mhecomodel/

[11] Poleshuck E, Perez-Diaz W, Wittink M, ReQua M, Harrington A, Katz J, et al. Resilience in the midst of chaos: Socioecological model applied to women with depressive symptoms and socioeconomic disadvantage. J Community Psychol. 2019;47(5):1000–13. doi: 10.1002/jcop.22188 30999386 PMC6944280

[12] Compton MT, Shim RS. The Social Determinants of Mental Health. Focus. 2015;13(4):419–25. doi: 10.1176/appi.focus.20150017

[13] Labonté R, Stuckler D. The rise of neoliberalism: how bad economics imperils health and what to do about it. J Epidemiol Community Health. 2016;70(3):312–8. doi: 10.1136/jech-2015-206295 26424847

[14] Frasquilho D, Matos MG, Salonna F, Guerreiro D, Storti CC, Gaspar T, et al. Mental health outcomes in times of economic recession: a systematic literature review. BMC Public Health. 2016;16:115. doi: 10.1186/s12889-016-2720-y 26847554 PMC4741013

[15] Suhrcke M, Stuckler D. Will the recession be bad for our health? It depends. Soc Scie Med. 2012;74(5):647–53. doi: 10.1016/j.socscimed.2011.12.011 22226605

[16] Catalano R, Goldman-Mellor S, Saxton K, Margerison-Zilko C, Subbaraman M, LeWinn K, et al. The health effects of economic decline. Annu Rev Public Health. 2011;32:431–50. doi: 10.1146/annurev-publhealth-031210-101146 21054175 PMC3855327

[17] Mercado CI, Bullard KM, Bolduc MLF, Andrews CA, Freggens ZRF, Liggett G, et al. A Shift in Approach to Addressing Public Health Inequities and the Effect of Societal Structural and Systemic Drivers on Social Determinants of Health. Public Health Rep. 2024:333549241283586. doi: 10.1177/00333549241283586 39394663 PMC11556650

[18] Alegría M, Alvarez K, Cheng M, Falgas-Bague I. Recent Advances on Social Determinants of Mental Health: Looking Fast Forward. Am J Psychiatry. 2023;180(7):473–82. doi: 10.1176/appi.ajp.20230371 37392038 PMC12096341

[19] Brown AF, Ma GX, Miranda J, Eng E, Castille D, Brockie T, et al. Structural Interventions to Reduce and Eliminate Health Disparities. Am J Public Health. 2019;109(S1):S72–8. doi: 10.2105/AJPH.2018.304844 30699019 PMC6356131

[20] Frieden TR. A Framework for Public Health Action: The Health Impact Pyramid. Am J Public Health. 2010;100(4):590–5. doi: 10.2105/ajph.2009.185652 20167880 PMC2836340

[21] Carey G, Crammond B. Systems change for the social determinants of health. BMC Public Health. 2015;15:662. doi: 10.1186/s12889-015-1979-8 26168785 PMC4501117

[22] Centers for Disease Control and Prevention (CDC). Behavioral Risk Factor Surveillance System survey questionnaire. 2019. Available from: https://www.cdc.gov/brfss/questionnaires/index.htm

[23] Greenlund KJ, Lu H, Wang Y, Matthews KA, LeClercq JM, Lee B, et al. PLACES: Local Data for Better Health. Prev Chronic Dis. 2022;19:E31. doi: 10.5888/pcd19.210459 35709356 PMC9258452

[24] Luchman JN. Determining relative importance in Stata using dominance analysis: domin and domme. Stata J. 2021;21(2):510–38. doi: 10.1177/1536867x211025837

[25] Galobardes B, Shaw M, Lawlor DA, Lynch JW, Davey Smith G. Indicators of socioeconomic position (part 1). J Epidemiol Community Health. 2006;60(1):7–12. doi: 10.1136/jech.2004.023531 16361448 PMC2465546

[26] Ridley M, Rao G, Schilbach F, Patel V. Poverty, depression, and anxiety: Causal evidence and mechanisms. Science. 2020;370(6522):eaay0214. doi: 10.1126/science.aay0214 33303583

[27] Santiago CD, Wadsworth ME, Stump J. Socioeconomic status, neighborhood disadvantage, and poverty-related stress: Prospective effects on psychological syndromes among diverse low-income families. J Econ Psychol. 2011;32(2):218–30. doi: 10.1016/j.joep.2009.10.008

[28] Sareen J, Afifi TO, McMillan KA, Asmundson GJG. Relationship between household income and mental disorders: findings from a population-based longitudinal study. Arch Gen Psychiatry. 2011;68(4):419–27. doi: 10.1001/archgenpsychiatry.2011.15 21464366

[29] Thomson RM, Igelström E, Purba AK, Shimonovich M, Thomson H, McCartney G, et al. How do income changes impact on mental health and wellbeing for working-age adults? A systematic review and meta-analysis. Lancet Public Health. 2022;7(6):e515–28. doi: 10.1016/S2468-2667(22)00058-5 35660213 PMC7614874

[30] Reiss F. Socioeconomic inequalities and mental health problems in children and adolescents: a systematic review. Soc Sci Med. 2013;90:24–31. doi: 10.1016/j.socscimed.2013.04.026 23746605

[31] Social Security Administration (SSA). SSI annual statistical report, 2019. SSA publication no 13-11827. 2020. Washington, DC: Office of Research, Evaluation, and Statistics. Available from: https://www.ssa.gov/policy/docs/statcomps/ssi_asr/2019/ssi_asr19.pdf

[32] Cree RA, Okoro CA, Zack MM, Carbone E. Frequent Mental Distress Among Adults, by Disability Status, Disability Type, and Selected Characteristics - United States, 2018. MMWR Morb Mortal Wkly Rep. 2020;69(36):1238–43. doi: 10.15585/mmwr.mm6936a2 32914770 PMC7499832

[33] Carlson S, Keith-Jennings B, Chaudhry R. SNAP Provides Needed Food Assistance to Millions of People with Disabilities. 2017. Available from https://www.cbpp.org/research/snap-provides-needed-food-assistance-to-millions-of-people-with-disabilities

[34] Gregory CA, Deb P. Does SNAP improve your health? Food Policy. 2015;50:11–9. doi: 10.1016/j.foodpol.2014.09.010

[35] Bureau of Labor Statistics (BLS). Current population survey. 2020. Available from: https://www.bls.gov/cps/tables.htm

[36] Demange PA, Boomsma DI, van Bergen E, Nivard MG. Evaluating the causal relationship between educational attainment and mental health. medRxiv. 2023. doi: 10.1101/2023.01.26.23285029 36747639 PMC9901051

[37] Phillips JA, Hempstead K. Differences in U.S. Suicide Rates by Educational Attainment, 2000-2014. Am J Prev Med. 2017;53(4):e123–30. doi: 10.1016/j.amepre.2017.04.010 28756896

[38] Breslau J, Lane M, Sampson N, Kessler RC. Mental disorders and subsequent educational attainment in a US national sample. J Psychiatr Res. 2008;42(9):708–16. doi: 10.1016/j.jpsychires.2008.01.016 18331741 PMC2748981

[39] Ferenchak NN, Katirai M. Commute mode and mental health in major metropolitan areas. Transportation Letters. 2014;7(2):92–103. doi: 10.1179/1942787514y.0000000040

[40] Kitagawa R, Kuroda S, Okudaira H, Owan H. Working from home and productivity under the COVID-19 pandemic: Using survey data of four manufacturing firms. PLoS One. 2021;16(12):e0261761. doi: 10.1371/journal.pone.0261761 34941956 PMC8700052

[41] Graif C, Arcaya MC, Diez Roux AV. Moving to opportunity and mental health: Exploring the spatial context of neighborhood effects. Soc Sci Med. 2016;162:50–8. doi: 10.1016/j.socscimed.2016.05.036 27337349 PMC4969097

[42] Ludwig J, Duncan GJ, Gennetian LA, Katz LF, Kessler RC, Kling JR, et al. Long-Term Neighborhood Effects on Low-Income Families: Evidence from Moving to Opportunity. Am Econ Rev. 2013;103(3):226–31. doi: 10.1257/aer.103.3.226

[43] Turney K, Kissane R, Edin K. After Moving to Opportunity. Soc Ment Health. 2012;3(1):1–21. doi: 10.1177/2156869312464789

[44] Thomas L. Hospitals, doctors moving out of cities to more affluent areas. Pittsburgh Post Gazette. 2014. Available from: https://archive.jsonline.com/news/health/hospitals-doctors-moving-out-of-poor-city-neighborhoods-to-more-affluent-areas-b99284882z1-262899701.html

[45] McKenzie TL, Moody JS, Carlson JA, Lopez NV, Elder JP. Neighborhood Income Matters: Disparities in Community Recreation Facilities, Amenities, and Programs. J Park Recreat Admi. 2013;31(4):12–22. 25006598 PMC4082954

[46] Venkataramani AS, O’Brien R, Whitehorn GL, Tsai AC. Economic influences on population health in the United States: Toward policymaking driven by data and evidence. PLoS Med. 2020;17(9):e1003319. doi: 10.1371/journal.pmed.1003319 32877406 PMC7467305

[47] Montez JK, Beckfield J, Cooney JK, Grumbach JM, Hayward MD, Koytak HZ, et al. US State Policies, Politics, and Life Expectancy. Milbank Q. 2020;98(3):668–99. doi: 10.1111/1468-0009.12469 32748998 PMC7482386

[48] Lakerveld J, Mackenbach J. The Upstream Determinants of Adult Obesity. Obes Facts. 2017;10(3):216–22. doi: 10.1159/000471489 28564658 PMC5644962

[49] Williams DR, Costa MV, Odunlami AO, Mohammed SA. Moving upstream: how interventions that address the social determinants of health can improve health and reduce disparities. J Public Health Manag Pract. 2008;14 Suppl(Suppl):S8–17. doi: 10.1097/01.PHH.0000338382.36695.42 18843244 PMC3431152

[50] DoreEC, Livingston IiiMD3rd, ShaferPR. Easing Cash Assistance Rules During COVID-19 Was Associated With Reduced Days Of Poor Physical And Mental Health. Health Aff (Millwood). 2022;41(11):1590–7. doi: 10.1377/hlthaff.2022.00740 36343321

[51] Berkowitz SA, Basu S. Unemployment Insurance, Health-Related Social Needs, Health Care Access, and Mental Health During the COVID-19 Pandemic. JAMA Intern Med. 2021;181(5):699–702. doi: 10.1001/jamainternmed.2020.7048 33252615 PMC8094006

[52] O’Campo P, Molnar A, Ng E, Renahy E, Mitchell C, Shankardass K, et al. Social welfare matters: a realist review of when, how, and why unemployment insurance impacts poverty and health. Soc Sci Med. 2015;132:88–94. doi: 10.1016/j.socscimed.2015.03.025 25795992

[53] Wilson N, McDaid S. The mental health effects of a Universal Basic Income: A synthesis of the evidence from previous pilots. Soc Sci Med. 2021;287:114374. doi: 10.1016/j.socscimed.2021.114374 34534779

[54] Kuroki M. State minimum wage and mental health in the United States: 2011–2019. SSM - Mental Health. 2021;1:100040. doi: 10.1016/j.ssmmh.2021.100040

[55] Paul Leigh J, Leigh WA, Du J. Minimum wages and public health: A literature review. Prev Med. 2019;118:122–34. doi: 10.1016/j.ypmed.2018.10.005 30316876

[56] Shields-Zeeman L, Collin DF, Batra A, Hamad R. How does income affect mental health and health behaviours? A quasi-experimental study of the earned income tax credit. J Epidemiol Community Health. 2021;75(10):929–35. doi: 10.1136/jech-2020-214841 33990398

[57] Boyd-Swan C, Herbst CM, Ifcher J, Zarghamee H. The earned income tax credit, mental health, and happiness. Journal of Economic Behavior & Organization. 2016;126:18–38. doi: 10.1016/j.jebo.2015.11.004

[58] Yearby R. When Equal Pay Is Not Enough: The Influence of Employment Discrimination on Health Disparities. Public Health Rep. 2019;134(4):447–50. doi: 10.1177/0033354919847743 31112455 PMC6598139

[59] Bovell-Ammon A, Yentel D, Koprowski M, Wilkinson C, Sandel M. Housing Is Health: A Renewed Call for Federal Housing Investments in Affordable Housing for Families With Children. Academic Pediatrics. 2021;21(1):19–23. doi: 10.1016/j.acap.2020.06.141 32619545

[60] Keith-Jennings B, Llobrera J, Dean S. Links of the Supplemental Nutrition Assistance Program With Food Insecurity, Poverty, and Health: Evidence and Potential. Am J Public Health. 2019;109(12):1636–40. doi: 10.2105/AJPH.2019.305325 31693420 PMC6836787

[61] Courtin E, Kim S, Song S, Yu W, Muennig P. Can Social Policies Improve Health? A Systematic Review and Meta-Analysis of 38 Randomized Trials. Milbank Q. 2020;98(2):297–371. doi: 10.1111/1468-0009.12451 32191359 PMC7296440

[62] Hahn RA, Truman BI. Education Improves Public Health and Promotes Health Equity. Int J Health Serv. 2015;45(4):657–78. doi: 10.1177/0020731415585986 25995305 PMC4691207

[63] Wray CM, Khare M, Keyhani S. Access to Care, Cost of Care, and Satisfaction With Care Among Adults With Private and Public Health Insurance in the US. JAMA Netw Open. 2021;4(6):e2110275. doi: 10.1001/jamanetworkopen.2021.10275 34061204 PMC8170543

[64] Robertson-Preidler J, Trachsel M, Johnson T, Biller-Andorno N. The Affordable Care Act and Recent Reforms: Policy Implications for Equitable Mental Health Care Delivery. Health Care Anal. 2020;28(3):228–48. doi: 10.1007/s10728-020-00391-0 32103383

[65] Fox A, Poirier R. How Single-payer Stacks Up: Evaluating Different Models of Universal Health Coverage on Cost, Access, and Quality. Int J Health Serv. 2018;48(3):568–85. doi: 10.1177/0020731418779377 29925286

[66] Oakman J, Kinsman N, Stuckey R, Graham M, Weale V. A rapid review of mental and physical health effects of working at home: how do we optimise health? BMC Public Health. 2020;20(1):1825. doi: 10.1186/s12889-020-09875-z 33256652 PMC7703513

[67] World Health Organization (WHO). Health in All Policies (HiAP): Helsinki Statement - Framework for country action. 2014. Available from: https://apps.who.int/iris/bitstream/handle/10665/112636/9789241506908_eng.pdf

[68] Alegría M, NeMoyer A, Falgàs Bagué I, Wang Y, Alvarez K. Social Determinants of Mental Health: Where We Are and Where We Need to Go. Curr Psychiatry Rep. 2018;20(11):95. doi: 10.1007/s11920-018-0969-9 30221308 PMC6181118

[69] Phelan JC, Link BG. Is Racism a Fundamental Cause of Inequalities in Health? Annu Rev Sociol. 2015;41(1):311–30. doi: 10.1146/annurev-soc-073014-112305

[70] Kämpfen F, Kohler IV, Ciancio A, Bruine de Bruin W, Maurer J, Kohler H-P. Predictors of mental health during the Covid-19 pandemic in the US: Role of economic concerns, health worries and social distancing. PLoS One. 2020;15(11):e0241895. doi: 10.1371/journal.pone.0241895 33175894 PMC7657497

[71] Kim D. Financial hardship and social assistance as determinants of mental health and food and housing insecurity during the COVID-19 pandemic in the United States. SSM Popul Health. 2021;16:100862. doi: 10.1016/j.ssmph.2021.100862 34692973 PMC8517203

[72] Lu X, Lin Z. COVID-19, Economic Impact, Mental Health, and Coping Behaviors: A Conceptual Framework and Future Research Directions. Front Psychol. 2021;12:759974. doi: 10.3389/fpsyg.2021.759974 34899503 PMC8660126

