# Supplementary material for: Economic factors associated with county-level mental health – United States, 2019
Source: PLoS One. 2025 Jun 4;20(6):e0300939. doi: 10.1371/journal.pone.0300939 (PMC12136295; doi:10.1371/journal.pone.0300939)
Supplement: S1 Fig — (a) In the overall model, the “elbow” on the plot at variable 12 (indicated in red) shows where the dominance weights drop off most steeply in value; values to the left of the elbow (11 total) were retained as significant for our overall linear regression model. (b) The urban scree plot indicates a steep drop in weights after variable 2, and the second steepest drop after variable 7. To capture additional factors impacting mental health, we retained the top seven variables for the urban linear regression model. (c) The steepest decline in the rural scree plot came after variable 8. The top eight variables were retained for the rural linear regression model. (DOCX) [file pone.0300939.s001.docx]

1. **Overall**


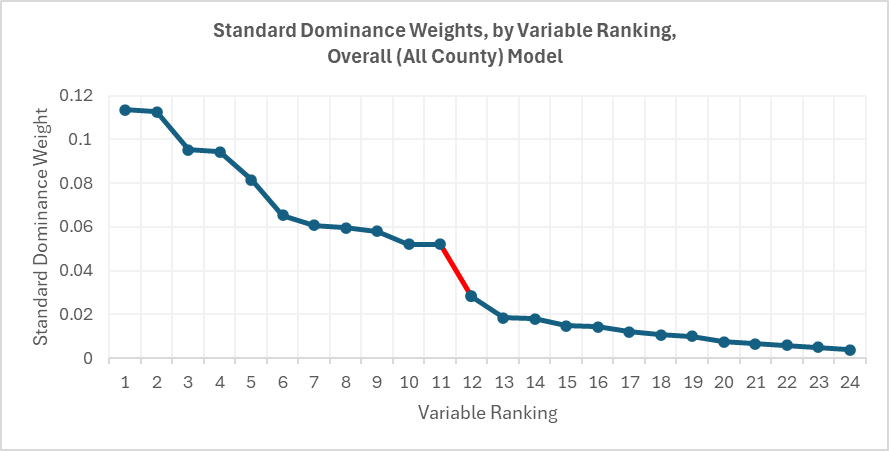


| **No.** | **Variable** | **Standardized Dominance Weight** |
| --- | --- | --- |
| 1 | Median household income | 0.1136 |
| 2 | Households with Supplemental Security Income (SSI) | 0.1126 |
| 3 | Population ≥ 25 years old with college degree | 0.0952 |
| 4 | Households with Supplemental Nutrition Assistance Program (SNAP) benefits in the  past 12 months | 0.0944 |
| 5 | Population with public health insurance coverage alone (Medicaid, Medicare, Veterans Administration [VA]) | 0.0815 |
| 6 | Unemployment rate, ≥16 years old | 0.0652 |
| 7 | Employees ≥ 16 years old working from home | 0.0607 |
| 8 | Median home value | 0.0597 |
| 9 | Households with Social Security income | 0.0580 |
| 10 | Mean travel time to work | 0.0521 |
| 11 | Employed but under the Federal Poverty Limit (FPL) (working poverty rate) | 0.0520 |
| 12 | Gini Index of income inequality | 0.0280 |
| 13 | Population 19-64 years old without health coverage | 0.0185 |
| 14 | 10-year population change | 0.0181 |
| 15 | Ratio of population to number of Primary Care Providers | 0.0147 |
| 16 | Real Gross Domestic Product (GDP) | 0.0144 |
| 17 | Homeownership | 0.0121 |
| 18 | Prevalence of rent-paying units with Gross Rent as a Percentage of Household Income (GRAPI) of 30% or more | 0.0106 |
| 19 | Prevalence of units with a mortgage with Selected Monthly Owner Costs as a Percentage of Household Income (SMOCAPI) of 30% or more | 0.0099 |
| 20 | Households with public cash assistance | 0.0074 |
| 21 | 10-year change in GDP, 2010 – 2019 | 0.0066 |
| 22 | Mean usual hours per week worked in the past 12 months for workers 16-64 years old | 0.0059 |
| 23 | Ratio of population to number of Mental Health Providers | 0.0049 |
| 24 | Female pay as a percentage of male pay | 0.0038 |

1. **Urban**


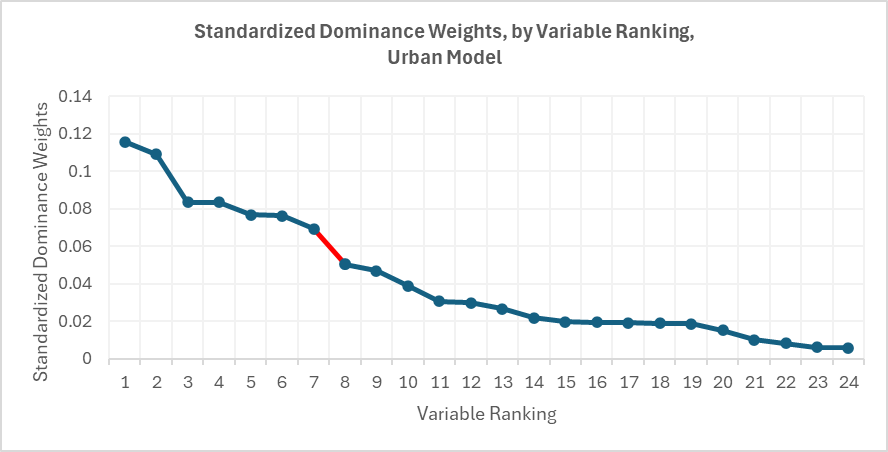


| **No.** | **Variable** | **Standardized Dominance Weight** |
| --- | --- | --- |
| 1 | Median household income | 0.1157 |
| 2 | Households with Supplemental Security Income (SSI) | 0.1090 |
| 3 | Population ≥ 25 years old with college degree | 0.0834 |
| 4 | Households with Supplemental Nutrition Assistance Program (SNAP) benefits in the  past 12 months | 0.0833 |
| 5 | Population with public health insurance coverage alone (Medicaid, Medicare, Veterans Administration [VA]) | 0.0768 |
| 6 | Households with Social Security income | 0.0763 |
| 7 | Median home value | 0.0693 |
| 8 | Employees ≥ 16 years old working from home | 0.0501 |
| 9 | Employed but under the Federal Poverty Limit (FPL) (working poverty rate) | 0.0470 |
| 10 | Unemployment rate, ≥16 years old | 0.0388 |
| 11 | Mean travel time to work | 0.0305 |
| 12 | 10-year population change | 0.0299 |
| 13 | Prevalence of units with a mortgage with Selected Monthly Owner Costs as a Percentage of Household Income (SMOCAPI) of 30% or more | 0.0265 |
| 14 | Real Gross Domestic Product (GDP) | 0.0219 |
| 15 | Female pay as a percentage of male pay | 0.0197 |
| 16 | Population 19-64 years old without health coverage | 0.0193 |
| 17 | Homeownership | 0.0192 |
| 18 | Gini Index of income inequality | 0.0189 |
| 19 | 10-year change in GDP, 2010 – 2019 | 0.0187 |
| 20 | Ratio of population to number of Primary Care Providers | 0.0152 |
| 21 | Households with public cash assistance | 0.0101 |
| 22 | Ratio of population to number of Mental Health Providers | 0.0083 |
| 23 | Prevalence of rent-paying units with Gross Rent as a Percentage of Household Income (GRAPI) of 30% or more | 0.0062 |
| 24 | Mean usual hours per week worked in the past 12 months for workers 16-64 years old | 0.0057 |

1. **Rural**


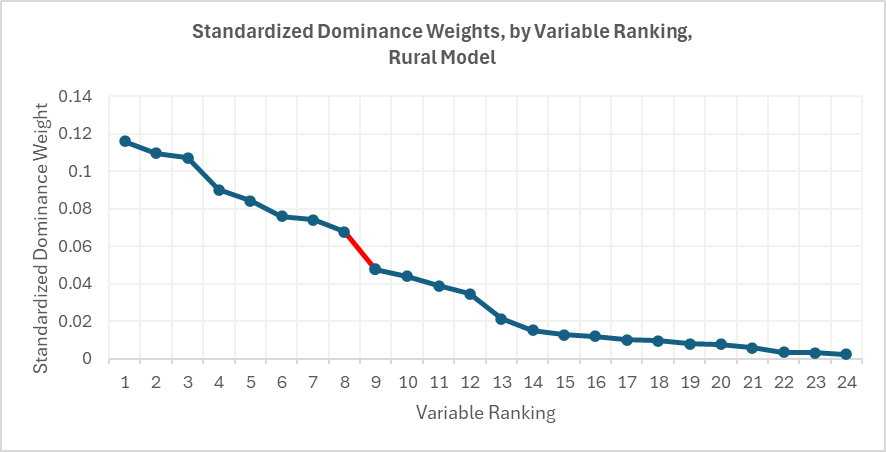


| **No.** | **Variable** | **Standardized Dominance Weight** |
| --- | --- | --- |
| 1 | Median household income | 0.1160 |
| 2 | Population ≥ 25 years old with college degree | 0.1096 |
| 3 | Households with Supplemental Security Income (SSI) | 0.1072 |
| 4 | Households with Supplemental Nutrition Assistance Program (SNAP) benefits in the  past 12 months | 0.0899 |
| 5 | Mean travel time to work | 0.0841 |
| 6 | Population with public health insurance coverage alone (Medicaid, Medicare, Veterans Administration [VA]) | 0.0758 |
| 7 | Unemployment rate, ≥16 years old | 0.0739 |
| 8 | Employees ≥ 16 years old working from home | 0.0677 |
| 9 | Employed but under the Federal Poverty Limit (FPL) (working poverty rate) | 0.0475 |
| 10 | Median home value | 0.0440 |
| 11 | Households with Social Security income | 0.0389 |
| 12 | Gini Index of income inequality | 0.0346 |
| 13 | Prevalence of rent-paying units with Gross Rent as a Percentage of Household Income (GRAPI) of 30% or more | 0.0213 |
| 14 | Population 19-64 years old without health coverage | 0.0151 |
| 15 | Ratio of population to number of Primary Care Providers | 0.0127 |
| 16 | Mean usual hours per week worked in the past 12 months for workers 16-64 years old | 0.0120 |
| 17 | Homeownership | 0.0100 |
| 18 | Prevalence of units with a mortgage with Selected Monthly Owner Costs as a Percentage of Household Income (SMOCAPI) of 30% or more | 0.0095 |
| 19 | Households with public cash assistance | 0.0080 |
| 20 | Real Gross Domestic Product (GDP) | 0.0076 |
| 21 | 10-year population change | 0.0057 |
| 22 | 10-year change in GDP, 2010 – 2019 | 0.0034 |
| 23 | Ratio of population to number of Mental Health Providers | 0.0032 |
| 24 | Female pay as a percentage of male pay | 0.0022 |
