# Supplementary material for: Economic factors associated with county-level mental health – United States, 2019
Source: PLoS One. 2025 Jun 4;20(6):e0300939. doi: 10.1371/journal.pone.0300939 (PMC12136295; doi:10.1371/journal.pone.0300939)
Supplement: S1 Table — (DOCX) [file pone.0300939.s002.docx]

| **Factor** | **Variable** | **Data Source** |
| --- | --- | --- |
| Business | 10-year percent change in Gross Domestic  Product (GDP), 2010 – 2019 (%) | US Bureau of Economic Analysis, calculated using estimates published in 2021 (CAGDP2) |
|  | Real GDP, in thousands of chained dollars ($) | US Bureau of Economic Analysis 2019 estimates, published in 2021 (CAGDP9) |
|  | Change in Real GDP, 2010 – 2019 (%) | US Bureau of Economic Analysis, calculated using estimates published in 2021 (CAGDP9) |
| Employment | Unemployment rate, ≥16 years old (%) | ACS 2019 5-year estimates (S2301) |
|  | Employed but under the Federal Poverty Limit (FPL) (%) | ACS 2019 5-year estimates (S1701) |
|  | Mean usual hours worked in the past 12 months  for workers 16-64 years old | ACS 2019 5-year estimates (B23020) |
|  | Employees ≥ 16 years old working from home (%) | ACS 2019 5-year estimates (S0801) |
|  | Mean travel time to work (minutes) | ACS 2019 5-year estimates (S0801) |
| Income and Wealth | Median earnings ($) | ACS 2019 5-year estimates (S2411) |
|  | Median household income ($10,000 increments) | ACS 2019 5-year estimates (S1901) |
|  | Population ≥ 25 years old with college degree (%) | ACS 2019 5-year estimates (S1501) |
|  | Gini Index of income inequality (score 0-1) | ACS 2019 5-year estimates (B19083) |
|  | Female pay as a percentage of male pay (%) | ACS 2019 5-year estimates (S2412) |
|  | Households with Social Security income (%) | ACS 2019 5-year estimates (DP03) |
|  | Households with Supplemental Security Income (SSI) (%) | ACS 2019 5-year estimates (DP03) |
|  | Households with public cash assistance (%) | ACS 2019 5-year estimates (DP03) |
|  | Homeownership (%) | ACS 2019 5-year estimates (DP04) |
| Expenses | Prevalence of units with a mortgage with  Selected Monthly Owner Costs as a Percentage  of Household Income (SMOCAPI) of 30% or  more (%) | ACS 2019 5-year estimates (DP04) |
|  | Prevalence of rent-paying units with Gross Rent  as a Percentage of Household Income (GRAPI)  of 30% or more (%) | ACS 2019 5-year estimates (DP04) |
|  | Households with Supplemental Nutrition  Assistance Program (SNAP) benefits in the past  12 months (%) | ACS 2019 5-year estimates (DP03) |
|  | Population 19-64 years old without health coverage (%) | ACS 2019 5-year estimates (S2701) |
|  | Population with public health insurance coverage alone (Medicaid, Medicare, Veterans Administration [VA]) (%) | ACS 2019 5-year estimates (S2704) |
| Neighborhood | Median home value, in $10,000 increments ($) | ACS 2019 5-year estimates (DP04) |
|  | 10-year population change (%) | ACS 2019 5-year estimates (B01003) |
| Health Care Supply | Ratio of population to number of primary care providers | County Health Rankings 2022, from Area Health Resources Files/American Medical Association 2010-2019 |
|  | Ratio of population to number of mental health providers | County Health Rankings 2020, from CMS, National Provider Identification 2019 data |
| Dependent Variable | Prevalence of > 14 poor mental health days of the last 30 days, age-adjusted (%) | PLACES 2021, modeled from BRFSS 2019 |

NB: Loving County, TX, uses 2018 data for Hours Worked and Female/Male Pay Gap, 2017 data for Travel Time to Work

* ACS = American Community Survey, CMS = Centers for Medicaid & Medicare Services, BRFSS = Behavioral Risk Factor Surveillance System
